# Expression and Prognostic Significance of Macrophage Inflammatory Protein-3 Alpha and Cystatin A in Nasopharyngeal Carcinoma

**DOI:** 10.1155/2015/617143

**Published:** 2015-11-08

**Authors:** Minzhong Tang, Ningjiang Ou, Cheng Li, Aiying Lu, Jun Li, Liping Ma, Weiming Zhong, Jianquan Gao, Yuming Zheng, Yonglin Cai

**Affiliations:** ^1^Wuzhou Health System Key Laboratory for Nasopharyngeal Carcinoma Etiology and Molecular Mechanism, Wuzhou, Guangxi 543002, China; ^2^Department of Clinical Laboratory, Wuzhou Red Cross Hospital, Wuzhou, Guangxi 543002, China; ^3^Department of Pathology, Wuzhou Red Cross Hospital, Wuzhou, Guangxi 543002, China; ^4^Department of Radiation Oncology, Wuzhou Red Cross Hospital, Wuzhou, Guangxi 543002, China

## Abstract

This study aims to investigate the expression of macrophage inflammatory protein-3 alpha (MIP-3*α*) and cystatin A in nasopharyngeal carcinoma (NPC) and their association with clinical characteristics and prognosis. Primary tumor specimens from 114 NPC patients and associated clinical follow-up data were collected, and the expression of MIP-3*α* and cystatin A proteins was investigated by immunohistochemistry. Expression of MIP-3*α* was significantly associated with TNM stage in patients with NPC (*P* < 0.05). NPC patients with positive expression of MIP-3*α* exhibited shorter median overall survival (OS) and distant metastasis-free survival (DMFS), compared with patients with negative expression (OS: 50.5 months versus 59.0 months, *P* = 0.013; DMFS: 50.1 months versus 60.2 months, *P* = 0.003). NPC patients with positive expression of cystatin A exhibited shorter median OS, local recurrence-free survival (LRFS), and DMFS, compared with patients with negative expression (OS: 51.1 months versus 60.0 months, *P* = 0.004; LRFS: 54.5 months versus 59.5 months, *P* = 0.036; DMFS: 52.3 months versus 58.8 months, *P* = 0.036). Both MIP-3*α* and cystatin A overexpressions in NPC tumor tissues were strong independent factors of poor prognosis in NPC patients. MIP-3*α* and cystatin A expressions may be valuable prognostic markers in NPC patients.

## 1. Introduction

Recent studies demonstrated that chemokines may play important roles in tumor proliferation and metastasis. Chemokines may be divided into four categories (CC, CXC, CX_3_C, and XC), in accordance with the relative positions of conserved cysteine residues [1]. Macrophage inflammatory protein-3 alpha (MIP-3*α*), encoded by the* CCL20* gene, regulates leukocyte trafficking through lymphoid tissues and induces leukocyte migration into sites of inflammation [2]. Previous reports indicate that MIP-3*α* expression is increased at certain inflammatory sites and tumors, including hepatocellular carcinoma [3] and pancreatic carcinoma [4]. MIP-3*α* also interacts with its receptor, CCR6, to promote the growth, migration, and invasion of pancreatic cancer cells.

Impairment of the basement membrane is considered an important marker of cancer invasion, and degradation of the extracellular matrix by proteolytic enzymes is a critical step in cancer cell invasion and metastasis. The proteolytic enzymes of the extracellular matrix, lysosomal cysteine proteases such as cathepsins B and L, as well as their endogenous inhibitors, cystatins, for example, cystatin A (also known as stefin A), cystatin B, and cystatin C, may play important roles in cancer progression and metastasis [5].

To date, radiation therapy is the preferred treatment for nasopharyngeal carcinoma (NPC) without distant metastasis. The local control rate of NPC is close to 100% because of continuing progression in imaging techniques and radiotherapy and the application of concurrent chemoradiotherapy [6, 7]. However, distant metastasis after treatment is a common cause of treatment failure, with a 5-year distant metastasis rate of 20%–32% in NPC patients [8, 9]. Previously, we showed that MIP-3*α* and cystatin A may be used to predict short-term clinical outcome in patients with NPC [10]. In this study, we evaluated the expression of MIP-3*α* and cystatin A in NPC tissues and correlated their expression with clinical characteristics and prognosis.

## 2. Materials and Methods

### 2.1. Patients

A total of 114 NPC patients, hospitalized in Wuzhou Red Cross Hospital between February 2009 and May 2010, who were previously untreated and biopsy proven and with no evidence of distant metastasis were included prospectively in this study (82 male patients, 32 female patients; median age 48 years, range 26–76 years). Patients were not treated by radiotherapy or chemotherapy prior to the biopsy. Cancer stage was defined according to the Chinese 2008 staging system [11]. All patients underwent radical radiotherapy for NPC. The accumulated doses to gross primary tumor and involved neck lymph nodes were 68–74 Gy and 64–70 Gy, respectively, and the uninvolved areas received 50–56 Gy. Patients with stage I-II NPC were treated by radiotherapy alone, whereas, for patients with stages III-IVa, chemotherapy was added to radiotherapy. This study was approved by the Clinical Research Ethics Committee of Wuzhou Red Cross Hospital.

### 2.2. Immunohistochemistry

Primary antibodies, including rabbit anti-MIP-3*α* antibody (ab85032, polyclonal; Abcam, Cambridge, UK), mouse anti-CCR6 (ab93086, monoclonal; Abcam), and mouse anti-cystatin A antibody (ab10442, monoclonal; Abcam), were diluted to 1 : 400, 1 : 500, and 1 : 400, respectively. Briefly, tissue sections were deparaffinized and dehydrated with xylene and ethanol. Antigen retrieval was performed by microwave heating, and sections were then incubated with hydrogen peroxide for 10 min and blocked with normal serum for 10 min, followed by incubation with primary antibody for 1 h. Detection was performed using a streptavidin-HRP kit (Nichirei, Tokyo, Japan) and sections were developed with diaminobenzidine (DAB). Tissues from human liver cancer and thymus tissue were used as positive controls for MIP-3*α* and cystatin A, respectively. Negative control sections were treated similarly with phosphate-buffered saline (PBS) instead of primary antibody.

The immunostaining results were evaluated and scored independently by two pathologists without knowledge of the clinicopathological outcomes of the patients. MIP-3*α*, CCR6, and cystatin A staining results were scored as four levels according to the percentage of cytoplasmic and/or membrane positive cells in 10 high-power fields as follows: (−): less than 10%, (+): 11%–20%, (++): 21%–50%, and (+++): >50%. Positive expression of MIP-3*α*, CCR6, and cystatin A was defined as >10% tumor cells with positive staining, whereas negative expression of MIP-3*α*, CCR6, and cystatin A was defined as <10% positive staining tumor cells.

### 2.3. Statistical Analyses

The frequency distribution of categorical variables was tested using a Chi-square test. The overall survival (OS) was calculated from the first day of chemoradiotherapy until the date of death or until the date of the last follow-up (January 26, 2014). The local recurrence-free survival (LRFS) was calculated from the first day of chemoradiotherapy until the date of either primary lesions in the nasopharynx or regional lymph node recurrence or until the date of the last follow-up. Distant metastasis-free survival (DMFS) was calculated from the first day of chemoradiotherapy until the date of distant metastasis or until the date of the last follow-up. Univariate analysis of patient survival was performed using the Kaplan-Meier method and log-rank comparison to evaluate differences between the survival curves. Multivariate analysis was performed using the Cox proportional hazards regression model and a forward stepwise logistic regression approach. There was a statistical difference when the *P* value was <0.05. Statistical analysis was performed using SPSS software version 13.0 (Chicago, IL, USA).

## 3. Results

### 3.1. MIP-3*α* and Cystatin A Expression in NPC Tissues and Association with Clinicopathological Characteristics

**Figure 1:**
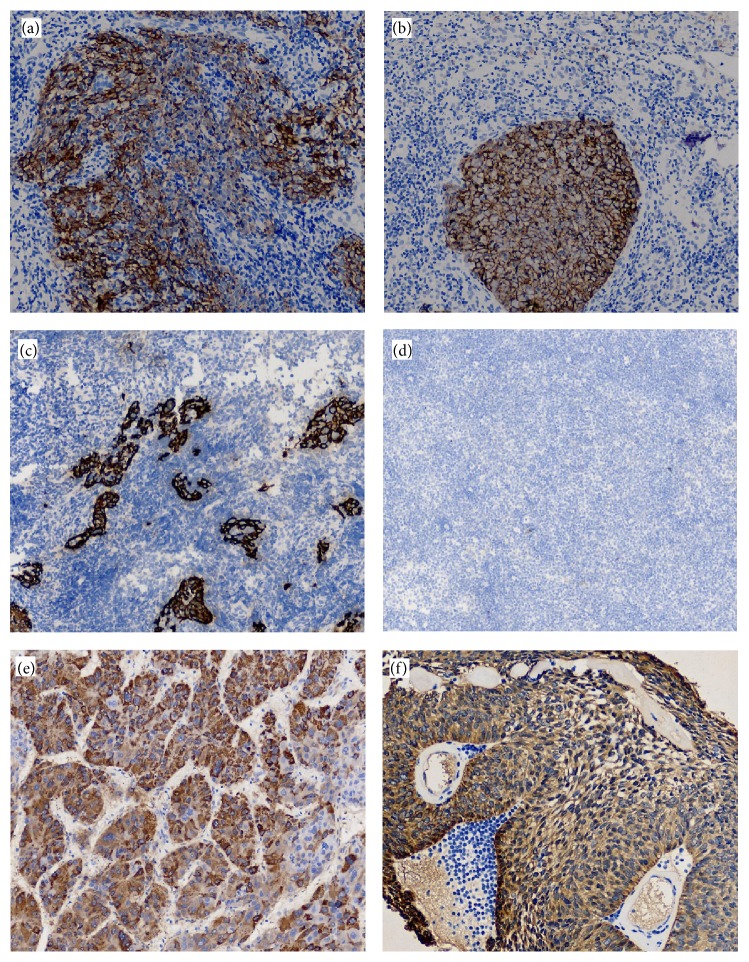
Immunohistochemistry staining of nasopharyngeal carcinoma (NPC) tissues (original magnification, ×200). (a) Positive expression of MIP-3*α* in NPC tissue. (b) Positive expression of cystatin A in NPC tissue. (c) Positive expression of CCR6 in NPC tissue. (d) Negative expression in NPC tissue as negative control. (e) Positive expression of MIP-3*α* in liver cancer tissue as positive control. (f) Positive expression of cystatin A in thymus tissue as positive control.

Immunohistochemical analysis of NPC tissues revealed that MIP-3*α* and cystatin A were predominantly expressed in the cytoplasm of cells in the cancer nests, and CCR6 was expressed in the cell membrane (Figure 1). MIP-3*α* was positively expressed in 57.9% (66/114) of tumor tissues. CCR6 was positively expressed in 44.7% (51/114) of tumor tissues. There was a significantly positive correlation in the expression of MIP-3*α* and CCR6 (*r* = 0.696, *P* < 0.001). MIP-3*α* expression was significantly higher in patients with stage III-IVa tumors compared with those with stage I-II tumors (*P* = 0.039; Table 1). In contrast, MIP-3*α* expression was not significantly associated with age, sex, pathologic type, tumor (T) classification, or lymph node (N) classification (*P* > 0.05; Table 1).

Cystatin A was positively expressed in 62.3% (71/114) of tumor tissues, and expression was associated with age (*P* = 0.009; Table 1), but not with sex, pathologic type, or stage (*P* > 0.05; Table 1).

### 3.2. Univariate Survival Analysis

**Figure 2:**
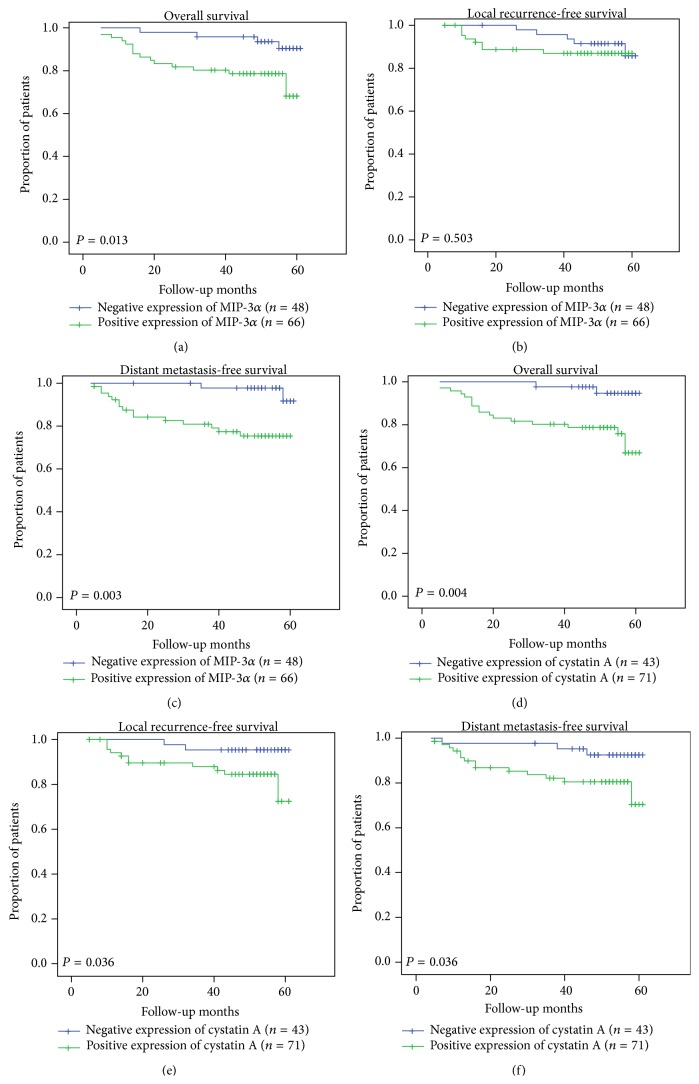
Kaplan-Meier curves of 114 NPC patients. Overall survival (a), local recurrence-free survival (b), and distant metastasis-free survival (c) of 114 NPC patients, stratified according to MIP-3*α* expression. Overall survival (d), local recurrence-free survival (e), and distant metastasis-free survival (f) of 114 NPC patients stratified according to cystatin A expression.

After a median follow-up of 53 months (range 5–61 months), the median OS, LRFS, and DMFS for all patients were 54.5 months, 56.6 months, and 54.9 months, respectively. The OS, LRFS, and DMFS curves for the positive and negative MIP-3*α* expression groups are shown in Figure 2. The median OS and DMFS were significantly poorer in patients with positive MIP-3*α* expression compared with patients with negative MIP-3*α* expression (OS: 50.5 months versus 59.0 months, *P* = 0.013, Figure 2(a); DMFS: 50.1 months versus 60.2 months, *P* = 0.003, Figure 2(c)). There was no statistical difference in median LRFS between the two groups (54.2 months versus 58.7 months, *P* = 0.503, Figure 2(b)).

The OS, LRFS, and DMFS curves for the positive and negative cystatin A expression groups are shown in Figure 2. The median OS, LRFS, and DMFS were significantly poorer in patients with positive cystatin A expression compared with patients with negative cystatin A expression (OS: 51.1 months versus 60.0 months, *P* = 0.004, Figure 2(d); LRFS: 54.5 months versus 59.5 months, *P* = 0.036, Figure 2(e); DMFS: 52.3 months versus 58.8 months, *P* = 0.036, Figure 2(f)).

### 3.3. Multivariate Analysis

The association of MIP-3*α* and cystatin A expression with OS, LRFS, and DMFS was examined further with Cox proportional hazards regression modeling, with adjustment for age, gender, WHO pathological classification, T classification, N classification, and chemotherapy. These analyses revealed that positive cystatin A expression, T classification, and N classification were independent prognostic factors of OS in NPC patients; T classification was an independent prognostic factor of LRFS; and age ≥ 45 years, N classification, and positive MIP-3*α* expression were also independent prognostic factors of DMFS (Table 2).

## 4. Discussion

Chemokines, which can direct cellular chemotactic movement, are low molecular weight cytokines secreted by various cell types. Most tumor cells express a wide range of chemokines and chemokine receptors and are in turn regulated by a complex network of chemokines and their associated receptors. Increased expression of MIP-3*α* has been reported in multiple myeloma [12], hepatocellular carcinoma [13], and oral cavity squamous cell carcinoma [14]. MIP-3*α*, through its CCR6 receptor, promotes tumor cell invasion in pancreatic adenocarcinoma [4]. In this study, we demonstrate that MIP-3*α* is positively expressed in 57.9% of NPC tissues. This suggests that NPC, like other cancers, exhibits high expression of MIP-3*α*. By analyzing the relationship between MIP-3*α* expression and clinical characteristics and prognostic factors, we found that patients with advanced stage NPC were more likely to have higher MIP-3*α* expression. Positive MIP-3*α* expression in patients with NPC was significantly associated with poorer OS and DMFS. MIP-3*α* is a powerful chemoattractant for T cells and immature dendritic cells, and regulatory T cells are recruited to NPC lesions to enhance local immunosuppression and help NPC cells evade antitumor immune responses. MIP-3*α* also appears to promote tumor cell proliferation and metastasis by attracting endothelial cells, accelerating angiogenesis, and affecting cancer cell mobility.

The ability to invade surrounding tissues and metastasize is an important feature of cancers. Penetration and degradation of components of the extracellular matrix and basement membrane are key steps in the metastatic process of cancer cells. The expression of specific proteolytic enzymes is triggered, and these enzymes are subsequently secreted and activated during the metastatic process. Proteolytic enzymes including serine proteases, matrix metalloproteinases, and lysosomal cysteine proteinase cathepsin B are strongly correlated with the metastatic state of cancer cells. The interaction between cathepsins and cystatins contributes to the degradation and remodeling of the extracellular matrix during cancer cell growth, angiogenesis, invasion, and metastasis [5]. Higher levels of cystatin A in body fluids have been associated with a poor prognosis in cancer patients [15]. In this study, positive cystatin A expression in patients with NPC was significantly associated with poorer OS, LRFS, and DMFS compared with those patients with negative cystatin A expression.

In multivariate analysis, we statistically weighted MIP-3*α* and cystatin A with clinicopathological characteristics to assess their relative prognostic impact. The tumor (T) classification and lymph node (N) classification remained powerful predictors of survival for NPC patients. MIP-3*α* and cystatin A were also independent prognostic factors.

## 5. Conclusion

Our results indicate that MIP-3*α* and cystatin A exhibit significant prognostic value in patients with NPC. Patients with positive expression of MIP-3*α* or cystatin A exhibited a poorer prognosis than those with negative expression of MIP-3*α* and cystatin A. On the basis of the presented results, additional studies are required (a) to evaluate the prognostic relevance of both factors in a more homogeneous and larger cohort of NPC patients; (b) to determine the value of their clinical applicability with respect to the selection of treatment in individual patients with this type of cancer; and (c) to investigate the mechanism of the two factors in the distant metastasis of NPC.

## Figures and Tables

**Table 1:** Correlation of expression of MIP-3*α* and cystatin A with clinical characteristics in 114 NPC patients [*n* (%)].

Characteristics	Number of patients	MIP-3*α*			Cystatin A		
		Negative	Positive	*P* value	Negative	Positive	*P* value
Age				0.197			0.009
<45 years	41	14	27		22	19	
≥45 years	73	34	39		21	52	
Gender				0.286			0.208
Male	82	32	50		28	54	
Female	32	16	16		15	17	
Pathology (WHO)^*∗*^							
Type I	1	0	1	0.952	0	1	0.722
Type II	12	5	7		4	8	
Type III	101	43	58		39	62	
T classification				0.145			0.100
T1-2	55	27	28		25	30	
T3-4	59	21	38		18	41	
N classification				0.117			0.861
N0-1	36	19	17		14	22	
N2-3	78	29	49		29	49	
Overall stage				0.039			0.181
I-II	15	10	5		8	7	
III-IVa	99	38	61		35	64	

^*∗*^Type I: keratinizing squamous cell carcinoma; type II: differentiated nonkeratinizing carcinoma; type III: undifferentiated nonkeratinizing carcinoma (according to World Health Organization histological classification).

**Table 2:** Multivariate analysis of prognostic factors using Cox proportional hazard ratio model.

Endpoint	Hazard ratio (95% CI)	*P* value
Overall survival		
T classification: T3-4 versus T1-2	4.66 (1.50–14.52)	0.008
N classification: N3 versus N0-2	4.16 (1.39–12.45)	0.011
Cystatin A: (+) versus (−)	4.93 (1.12–21.63)	0.035
Local recurrence-free survival		
T classification: T3-4 versus T1-2	12.99 (1.69–100.02)	0.014
Distant metastasis-free survival		
Age: ≥45 y versus <45 y	3.57 (1.07–11.89)	0.038
N classification: N3 versus N0-2	3.66 (1.12–12.02)	0.032
MIP-3*α*: (+) versus (−)	8.10 (1.82–35.93)	0.006

CI = confidence interval.
